# Intraspecies interactions of *Streptococcus mutans* impact biofilm architecture and virulence determinants in childhood dental caries

**DOI:** 10.1128/msphere.00778-23

**Published:** 2024-07-11

**Authors:** Stephanie S. Momeni, Xixi Cao, Baotong Xie, Katherine Rainey, Noel K. Childers, Hui Wu

**Affiliations:** 1 Department of Oral Rehabilitation and Biosciences, School of Dentistry, Oregon Health & Science University, Portland, Oregon, USA; 2 Department of Pediatric Dentistry, School of Dentistry, The University of Alabama at Birmingham, Birmingham, Alabama, USA; The University of Iowa, Iowa City, Iowa, USA

**Keywords:** *Streptococcus mutans*, intraspecies, biofilms, *Drosophila*

## Abstract

Early childhood dental caries (ECC) is the most common chronic disease among children, especially among low socioeconomic populations. *Streptococcus mutans* is most frequently associated with initiation of ECC. Although many studies report children with multiple *S. mutans* strains (i.e., genotypes) have greater odds of developing ECC, studies investigating intraspecies interactions in dental caries are lacking. This study investigates the impact of intraspecies interactions on cariogenic and fitness traits of clinical *S. mutans* isolates using *in vitro* and *in vivo* approaches. Association analysis evaluated if presence of multiple *S. mutans* genotypes within the first year of colonization was associated with caries. Initially, clinical *S. mutans* isolates from 10 children were evaluated. *S. mutans* strains (G09 and G18, most prevalent) isolated from one child were used for subsequent analysis. Biofilm analysis was performed for single and mixed cultures to assess cariogenic traits, including biofilm biomass, intra-polysaccharide, pH, and glucan. Confocal laser scanning microscopy (CLSM) and time-lapse imaging were used to evaluate spatial and temporal biofilm dynamics, respectively. A *Drosophila* model was used to assess colonization *in vivo*. Results showed the mean biofilm pH was significantly lower in co-cultured biofilms versus monoculture. Doubling of *S. mutans* biofilms was observed by CLSM and *in vivo* colonization in *Drosophila* for co-cultured *S. mutans*. Individual strains occupied specific domains in co-culture and G09 contributed most to increased co-culture biofilm thickness and colonization in *Drosophila*. Biofilm formation and acid production displayed distinct signatures in time-lapsed experiments. This study illuminates that intraspecies interactions of *S. mutans* significantly impacts biofilm acidity, architecture, and colonization.

**IMPORTANCE:**

This study sheds light on the complex dynamics of a key contributor to early childhood dental caries (ECC) by exploring intraspecies interactions of different *S. mutans* strains and their impact on cariogenic traits. Utilizing clinical isolates from children with ECC, the research highlights significant differences in biofilm architecture and acid production in mixed versus single genotype cultures. The findings reveal that co-cultured *S. mutans* strains exhibit increased cell density and acidity, with individual strains occupying distinct domains. These insights, enhanced by use of time-lapsed confocal laser scanning microscopy and a *Drosophila* model, offer a deeper understanding of ECC pathogenesis and potential avenues for targeted interventions.

## INTRODUCTION

Dental caries is a complex, multi-factorial disease, affecting the oral health and overall health of over 80% of the human population worldwide (1, 2). The Center for Disease Control and Prevention (https://www.cdc.gov/) lists dental caries as the most common chronic disease of children and adolescents. The World Health Organization reports that 60%–80% of school children have dental caries in industrialized countries and predicts developing nations will see an increase due to consumption of fermentable carbohydrates and lack of proper fluoridation (3). Early childhood caries is defined as having decayed, missing, or filled tooth surfaces (DMFS) in children under 6 years of age (4). Severe early childhood dental caries (SECC) is of particular concern, affecting the health and well-being of children under 6 years with extensive tooth decay. SECC is highly prevalent in children from marginalized groups such as minorities, individuals of low socioeconomic status, and those with limited access to dental care (4).

Many factors are thought to play a role in the disease initiation and progression, including the presence of specific oral bacteria. A primary focus in understanding the etiology of dental caries has been the search for microorganisms involved in the initiation, development, and progression of the disease. Research suggests that oral disease may evolve from major homeostatic imbalances in the oral microbial community. Under certain conditions, the ecology of the oral cavity selects for advantaged organisms that are conducive to a cariogenic state. Those dominate microorganisms include the mutans streptococci (MS), lactobacilli, *Actinomyces*, bifidobacteria, and yeasts (5, 6).

The primary bacteria typically associated with the initiation of early childhood dental caries are the MS, including *Streptococcus mutans* and *Streptococcus sobrinus* (7–9). In the case of SECC, numerous studies have reported that the primary infectious agent is *S. mutans* and the presence of *S. mutans* is widely considered the most effective predictor of future caries incidence (10). The mutans streptococci are involved in the initiation of caries, creating an environment conducive to aciduric organisms colonization and demineralization of tooth surfaces. *S. mutans* strains are highly diverse in their phenotypic characteristics and cariogenic potential (11, 12). Key pathogenic features include the ability to bind to teeth by formation of tenacious biofilms (i.e., dental plaque), to metabolize fermentable carbohydrates (especially sucrose) to produce organic acids, and to survive acidic environment (aciduricity) (11). Intracellular iodophilic polysaccharide (IPS) is an indicator of glycogen storage ability of *S. mutans* which allows for continued production of acid once carbohydrate sources are depleted (13).

Previously, we investigated *S. mutans* genetic diversity using different genotyping methods, repetitive extragenic palindromic-polymerase chain reaction (rep-PCR), multilocus sequence typing, serotyping by PCR, and whole genome sequencing (14–18). The scale and scope of the data from this 8-year longitudinal epidemiological study afforded a unique opportunity to determine the relationship between *S. mutans* genotypes and early childhood caries in a localized, high-caries risk population of African-American children (18). Children with higher *S. mutans* counts were 5.6 times more likely to develop caries and are significantly more likely to have higher caries scores (19). Children in our study population with multiple *S. mutans* genotypes are 4.5 times more likely to have dental caries experience, and younger children (age < 6 years) are 2.9 times more likely to have multiple genotypes (20). Others have reported similar findings linking the presence of more *S. mutans* genotypes with higher caries scores (21–27).

Despite literature supporting an association of intraspecies impact on caries potential, the majority of the research available has focused on evaluating either single or polymicrobial interspecies cultures or biofilms. Studies investigating the intraspecies interactions of multiple *S. mutans* genotypes and how these interactions contribute to caries virulence potential are lacking. In particular, studies investigating *S. mutans* intraspecies interactions in biofilms are crucial to examine how the presence of multiple *S. mutans* contribute to caries disease progression. Most studies investigating intraspecies interaction of *S. mutans* have largely focused on bacteriocins (i.e., mutacins in *S. mutans*) using stab agar antagonism assays and demonstrate that some *S. mutans* strains can have inhibitory effects on other *S. mutans* (28–30). This poses the question: is the presence of multiple *S. mutans* mutualistic or antagonistic in forming the cariogenic environment? Based on our previous association data for our study population, we hypothesized that the presence of multiple *S. mutans* genotypes contribute significantly to cariogenic traits and may provide a mutualistic benefit for *S. mutans* fitness. In this study, we investigate the impact of multiple *S. mutans* genotypes on cariogenic potential using *in vitro* (biofilm) and *in vivo* (*Drosophila* model) approaches.

## RESULTS

### Presence of multiple *S. mutans* genotypes within the first year of detection is significantly associated with caries

Based on the summary of longitudinal data (6 years), we previously reported that younger children (age < 6 years) within our study population were more likely to have multiple *S. mutans* genotypes and that there was a significant association between having multiple genotypes and dental caries (20). Here, we determine if having multiple *S. mutans* genotypes at initial detection of *S. mutans* or within the first follow-up visit after detection is associated with dental caries (DMFS score > 0 within 6 years); a cross-sectional analysis was performed. Children with multiple genotypes at initial detection were not significantly associated with developing caries. This is logical as there is a time lapse (typically 1–2 years) from initial colonization to development of detectable caries. However, the presence of multiple genotypes within the first year of detection was found to be significantly associated with developing ECC (Table 1), highlighting that early colonization with multiple *S. mutans* is a risk factor for caries. For children with multiple genotypes, the mean number of genotypes was 2.7 (range, two to six genotypes) within the first year of detection. The majority of children with mulitple genotypes had aquired these genotypes between 1–2.5 years of age. Of the 14 caries-free children in both groups (10 single or 4 multiple), 12 (86%) children did not maintain persistent colonization of *S. mutans* for the duration of the study (6 years).

**TABLE 1:** Presence of multiple *Streptococcus mutans* vs caries (*n* = 78)^*b*^

Number of genotypes		1	>1	
Initial detection of *S. mutans*	Caries	44 (56.4%)	20 (25.6%)	*P* = 0.327^*a*^
	No caries	12 (15.4%)	2 (2.6%)	
*S. mutans* within first year of detection	Caries	23 (29.5%)	41 (52.6%)	*P* = 0.019^*a*^
	No caries	10 (12.8%)	4 (5.1%)	

Distribution of genotypes within first year of *S. mutans* detection						
Number of genotypes	1	2	3	4	5	6
	33	28	9	4	3	1

Visit (approximate age) of children at first *S. mutans* detection							
	Baseline (1 year)	Visit 3 (1.5 years)	Visit 4 (2 years)	Visit 5 (2.5 years)	Visit 6 (3 years)	Visit 7 (3.5 years)	Visit 8 (4 years)
Single *S. mutans* strain	5	13	8	4	2	0	1
Multiple *S. mutans* strains	18	12	7	6	1	1	0

^*a*^ Fisher’s exact test.

^*b*^ Caries or no caries is based on a decayed, missing, filled, or surfaces score DMFS >0 at any time within the 6-year study.

### Biofilm pH is significantly lower in co-cultured *S. mutans* biofilms

**Fig 1:**
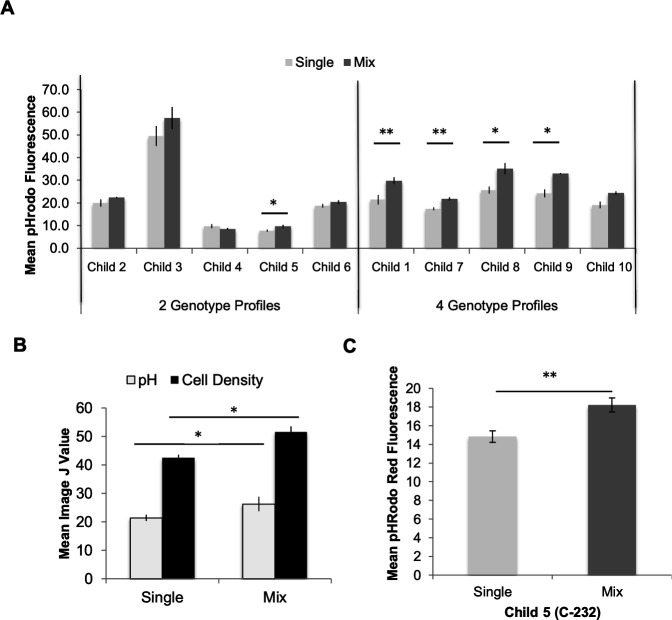
Impact of mono-cultured (single) vs co-cultured (mix) *S. mutans* on biofilm pH. (**A**) Children with mixed genotypes demonstrate a trend of decreased mean biofilm pH in mixed versus single strain cultures from 9 out of 10 children. Lower pH is shown by increased fluorescence of pHrodo dextran-conjugated probe. Mean fluorescence data obtained using ImageJ. Results are from a single experiment with three technical replicates to show the population trend. (**B**) Overall mean biofilm for all 10 children shows significantly lower biofilm pH and higher cell density by mean ImageJ analysis of fluorescence. (**C**) *S. mutans* from Child 5 consistently demonstrates significantly lower biofilm pH for the mixed strains than single strains alone. Results from at least three independent experiments with three techinical replicates each. Isolates from Child 5 (C-232) were selected for continued study because it presented with G09 and G18, the most common genotypes observed in this high-caries risk population. Standard error bars shown. *P < 0.05, **P < 0.01.

To investigate the effects of co-culture (mix) of different genotypes of *S. mutans* from the same individuals, we evaluated static biofilms for key cariogenic traits (biomass, pH, and IPS) for 10 randomly selected children with either two or four *S*. *mutans* genotypes. Co-cultured isolates from 9 out of 10 children showed a trend that co-culture biofilms have lower biofilm pH compared to mean mono-culture biofilms as indicated by increased fluorescence of the pHrodo probe (Fig. 1A). Child 5 (two genotypes) and Children 1, 7, 8, 9 (four genotypes) had significantly lower mean biofilm pH for the co-culture biofilms than combined mono-cultures. Overall mean biofilm pH for the population (10 children) of the co-cultures showed a significant increase in pHrodo fluorescence, indicating lower biofilm pH and significant greater cell density by increased Syto9 fluorescence (Fig. 1B). The latter is significant as it provides the first indication that co-cultured biofilms might be thicker. No overall significant differences in biofilm biomass or IPS was observed for the population (Fig. S1). For Child 5 (subject C-232), selected for subsequent analysis, the mean co-culture biofilm consistently had significantly lower biofilm pH than mono-culture (Fig. 1C).

**Fig 2:**
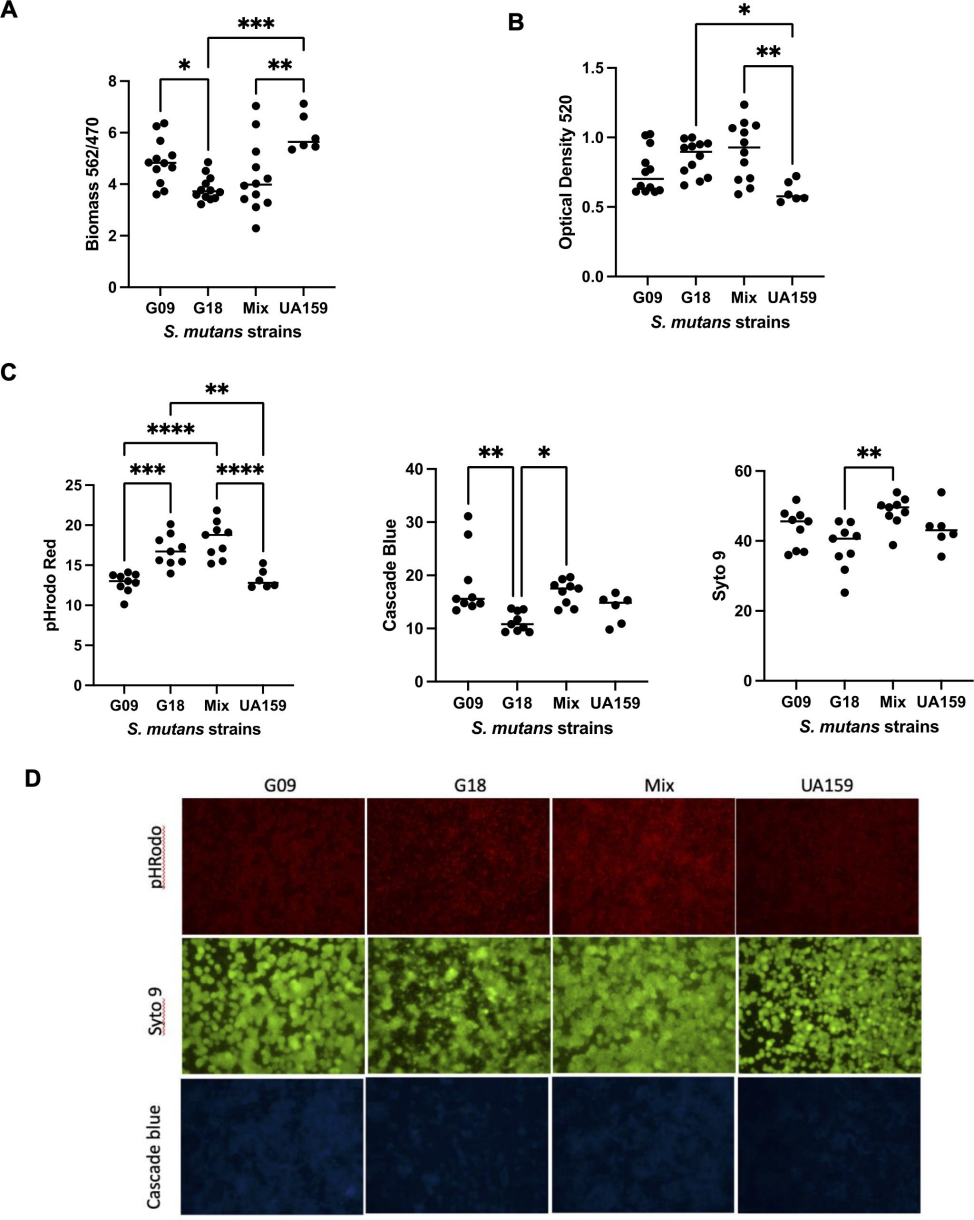
Effect of co-cultured *S. mutans* (mix of G09 and G18) versus mono-cultures (G09 or G18) on virulence properties using *in vitro* biofilms. Both G09 and G18 were from Child 5 (C-232). (**A**) Mean biofilm mass by crystal violet assay normalized to cell growth (OD_470_). (**B**) Mean intracellular polysaccharide by iodine assay (**C**) Mean ImageJ values for biofilm pH (pHRodo, red), glucan (cascade blue), and cell density (Syto9, green). Increase in pHrodo fluorescence indicates more acidic biofilms. (**D**) Representative images of biofilm pH (pHrodo red), glucan (cascade blue), and cell density (Syto9 green) shown with 10× magnification. Increased brightness in the mix (co-cultured) biofilm is significantly brighter. All biofilms were performed in Todd Hewitt broth + 1% sucrose. *N* = 3 independent experiments with a minimum of three technical replicates. *P < 0.05, **P < 0.01, and ***P < 0.001 by one way analysis of variance with post-hoc Tukey test.

Child 5 consisted of *S. mutans* genotypes G09 and G18, previously identified by rep-PCR (Fig. 2). No significant difference was observed in biomass or IPS between mono- and co-cultured strains, although the mix did trend higher for IPS. Biomass was significantly lower for G18 as compared to G09 mono-culture. Biomass for G18 mono-cultured and co-cultured biofilms was significantly less when compared to *S. mutans* UA159 control (Fig. 2A). This is consistent with previously reported data for the representative library G18 strain (UAB-10) (18). However, IPS was significantly higher for G18 mono-culture and co-cultured biofilms as compared to *S. mutans* UA159 (Fig. 2B), indicating greater glycogen storage in the clinical isolates.

For biofilm pH for Child 5, the G18 mono-culture and co-culture were significantly more acidic than the G09 mono-culture (indicated by increased fluorescence of pHrodo) (Fig. 2C and D). Both G18 mono-culture and co-cultured biofilms were significantly more acidic than *S. mutans* UA159, which is consistent with previously published findings for another G18 strain, UAB-10 (18). When the combined mean of the mono-cultures are compared to the mean co-cultured, the mix remains significantly more acidic (Fig. 1C). Cascade blue is a dextran-conjugated dye used to label extracellular glucans (Fig. 2C and D). Glucan for G18 was significantly lower when compared with both the G09 mono-culture and co-cultured biofilms. This reduction in glucans for G18 did not impact the pHrodo data for G18, indicating the acidic phenotype observed in co-culture was not due to variable glucan production between strains. Cell density (Syto9) in 2D imaging was consistently significantly higher in co-culture biofilm as compared to mono-cultured G18 biofilms indicating greater cell growth within the mix.

### Co-culturing *S. mutans* significantly increases *S. mutans* cell density and biofilm thickness by confocal laser scanning microscopy (CLSM)

**Fig 3:**
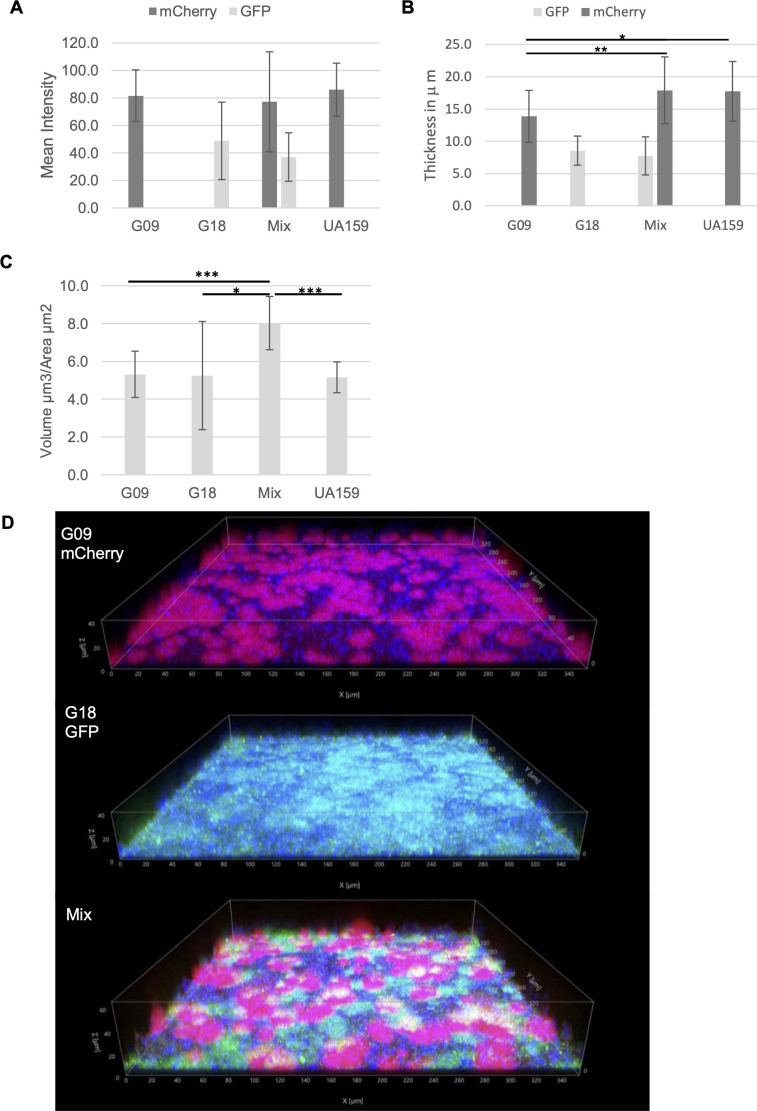
CLSM imaging and quantification of *S. mutans* single and mixed biofilms. (**A**) Mean fluorescence intensity for *S. mutans* by CLSM. (**B**) Mean *S. mutans* biofilm thickness by CLSM. Both fluorescence intensity and thickness show an increase in total *S. mutans* signal within the mix when both mCherry and GFP fluorescence is combined, though only G09 in the mix shows a significant difference in biofilm thickness. (**C**) Average volume/area demonstrates significant increase in area of *S. mutans* in mix versus single culture biofilms. Mean areas of mix for GFP and mCherry are added together. (**D**) CLSM image for single G09 (mCherry red), G18 (GFP green), and mix (red and green) illustrating shift in spatial architecture and biofilm heights of *S. mutans* in single vs mixed biofilms. Cascade blue was used to image extracellular glucan matrix. *N* = 3 with five to seven technical replicates per experiment. Note: Video image of single and mixed biofilm CLSM is available in supplemental materials online. *P < 0.05, **P < 0.01, and ***P < 0.001 by Student’s *t*-test (pairwise comparison of all samples with mix only).

To investigate the impact of co-culture on the biofilm thickness and architecture, *S. mutans* G09 and G18 were genetically modified with mCherry red and green fluorescent protein (GFP), respectively, for CLSM analysis. The mean fluorescence of mono- and co-cultured biofilms was comparable for individual strains, while G18 was significantly lower than UA159 (Fig. 3A). Even though all biofilms were initially inoculated with comparable concentractions of *S. mutans* (mix composed of 50% each strain, equal volume *S. mutans* to mono-cultures), when GFP and mCherry signals for the co-culture are considered together, total fluorescence is almost doubled, suggesting increased cell density in the mix. This was also observed when measuring biofilm thickness; however, G09 showed significantly greater biofilm thickness in the mix than its mono-culture (Fig. 3B). Both mono-cultures had significantly reduced thickness compared with UA159. The statistically significant doubling of *S. mutans* in the mix versus mono-cultures and UA159 is best illustrated with mean volume/area as this represents the total area of the fluorescence of *S. mutans* within the biofilm (Fig. 3C) since the areas of GFP and mCherry signals can be combined in this approach.

The shift in spatial arrangement and biofilm thickness in mixed biofilm vs mono-culture biofilms is illustrated in Fig. 3D and Movie S1. In mono-culture, G09 (red) forms large aggregates with noticeable glucan matrix (blue) forming the extracellular space between, while G18 demonstrates a confluent lawn phenotype with minimal visible glucans. However, in co-culture, G09 forms larger and taller aggregates (Fig. 3D and Fig. S2), which collapse inward on themselves, forming volcano-like structures with negative space in the underlying glucan layer (Movie S1). Interestingly, each strain occupies a specific domain with no apparent overlay.

It is noteworthy that the genetic modification with mCherry was not responsible for the aggregate phenotype observed in G09. A subsequent experiment of G09 with GFP displayed the same phenotype (not shown). However, labeling G18 with mCherry did promote an aggregate phenotype (data not shown), indicating that mCherry can alter biofilm formation in some clinical strains of *S. mutans*. Thus, the use of an alternate fluorescent protein is encouraged as a control when possible.

Given difference in pH, cell density, and thickness, it was crucial to confirm that the inoculum of *S. mutans* used was comparable between mono- and co-cultures at set-up. To evaluate this, G09 and G18 were genetically modified with different antibiotic resistance genes and differential plating was used at biofilm set-up and after biofilm formation. No significant difference in the total number of *S. mutans* used to inoculate the biofilms was observed using colony forming units (CFUs per milliliter) plating on antibiotic selective media for either pre-plate or post-plate (Fig. S3). The distributions of both strains within the co-culture biofilms both pre-plate and post-plate were comparable, with each comprising approximately half the total *S. mutans* in the mix.

### Different *S. mutans* clinical strains display distinguishable biofilms and acid formation phenotypes

Since co-cultured biofilms displayed a dramatic difference in biofilm structure, we next evaluated the formation of the biofilms using time-lapsed imaging for 24 hours. Using this approach, it was possible to observe biofilm formation dynamics for G09 and G18 as early as 3–4 hours (Fig. S4). Interestingly, biofilm formation was clearly distinguishable between the two *S*. *mutans* strains in single and co-cultured mix biofilms over the first 12 hours. This difference was best observed in the contrasted oblique illumination mode (Fig. S5; Movie S2); G09 displays dense microcolony-like mini-aggregates early in biofilm formation while G18 forms thinner, broader “fish net” networks of streptococccal chains. These distinct architectures are also observed in the fluorescence mode as the biofilm develops (Fig. S5 and Movie S2). As previously observed in the CLSM, the two strains occupy distinct domains within the biofilm with no appreciable overlap observed.

**Fig 4:**
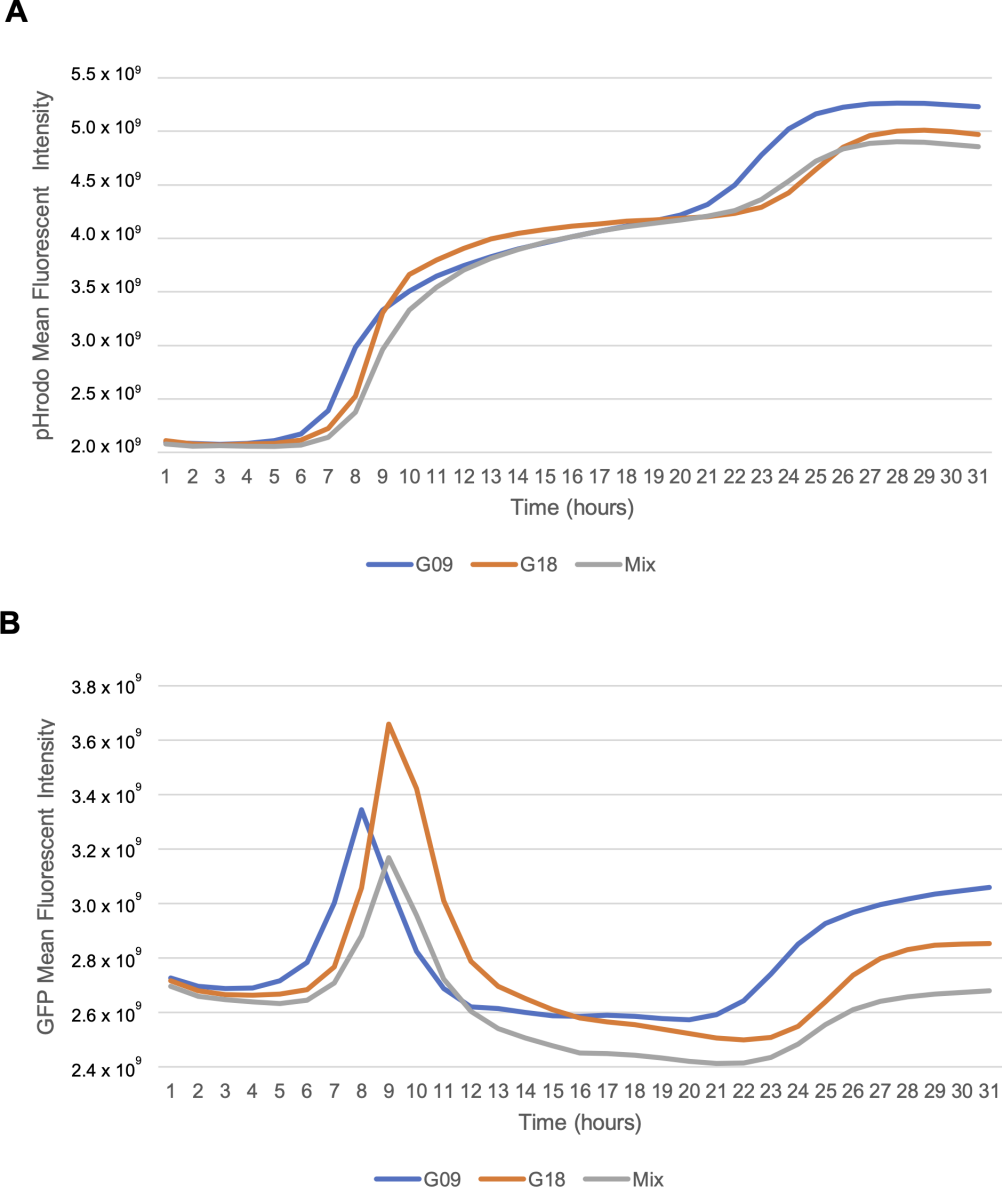
*Streptococcus mutans* biofilm pH displays two fluorescent peaks over time. (**A**) *S. mutans* biofilm pH analyzed with pHrodo red exhibits continuously increasing fluorescence with two fluorescent peaks over 30 hours. (**B**) *S. mutans* GFP cell fluorescence exhibits a sharp preliminary peak intensity before waning followed by a slow recovery. Time-lapse data were collected using a Zeiss Cell Discoverer7 (CD7) microscope with 50× PlanApp Water immersion lens. Mean data were collected for 41 z-stacks taken per hour per image site over three independent experiments performed in duplicate with three to four image sites per well. Source of *S. mutans* G09 and G18 is Child 5 (C-232).

Since biofilm formation was distinguishable between G09 and G18, we subsequently evaluated biofilm pH dynamics with pHrodo red using 30-hour time-lapse imaging with both strains labeled with GFP. Time was extended to 30 hours to better capture the second pH fluorescence increase. The pHrodo fluorescent intensity continually increased over time with two distinct phases at 10–11 hours and 24–26 hours (Fig. 4A). GFP fluorescence peaked between 8 and 9 hours, then decreased for several hours until GFP intensity partially recovered and plateaued around 25–26 hours (Fig. 4B). The decrease in GFP fluorescence may be due to GFP’s known sensitivity to low pH or *S. mutans* acid tolerance, but the overall biofilm adapts to the new aciduric environment. Fluorescent intensity of pHrodo consistently peaked within the 2 hours following the GFP fluorescence peaks.

### Co-culture of *S. mutans* improves *S. mutans* colonization *in vivo*

**Fig 5:**
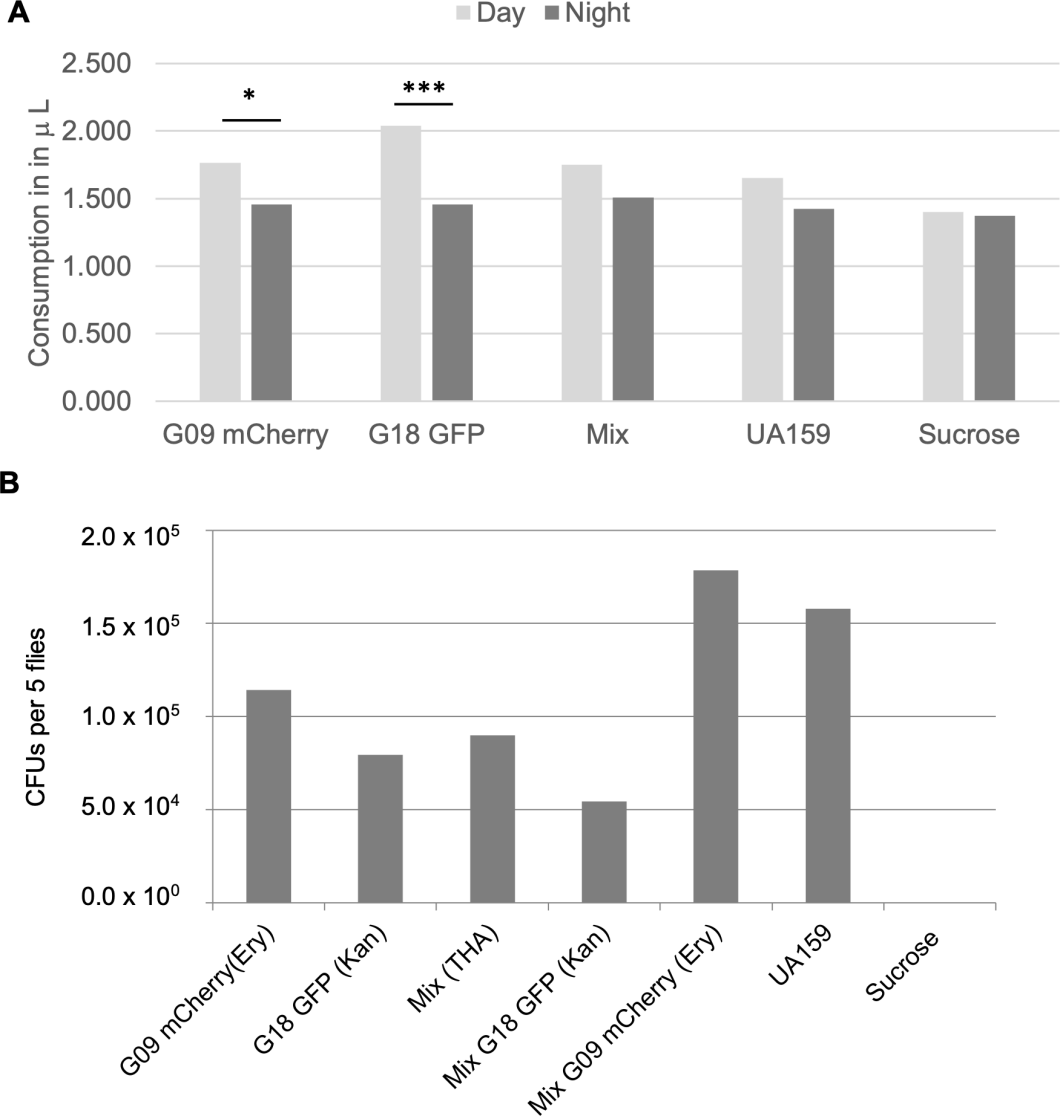
Mean *S. mutans* consumption and colonization in *Drosophila* with mono- and co-cultures. GFP, green fluorescent protein; mCherry, red fluorescent protein. (**A**) Normalized mean consumption of bacterial suspensions in 5% sucrose for seven flies over 3 days. Day = 8 hours, night = 16 hours/2. No significant difference was observed for the mean consumption over 3 days. UA159 and sucrose are positive and negative controls, respectively. (**B**) Mean CFUs per five flies differentially plated on Todd Hewitt agar with and without antibiotics show increased colonization of G09 with decreased G18 in the co-cultures as compared with mono-cultures. *N* = 3 with two technical replicates each. Error bars are not shown as variance is high between experiments; however, trend remains consistent. *P < 0.05, **P < 0.01, ***P < 0.001 by Student’s *t*-test.

A fly model was used to evaluate *in vivo* colonization of single or co-cultured *S. mutans (31–37*). Using the capillary feeding *Drosophila* model illustrates how co-cultures of *S. mutans* influence colonization within the *Drosophila* as compared to mono-cultures. The consumption of bacteria in 5% sucrose was comparable with no significant difference when comparing mean feedings for either day or night feeding across strains (Fig. 5A). There was a significant increase in day consumption for both single strains as compared to night feeding. *S. mutans* G09 colonization within *Drosophila* was consistently greater when G18 was also present (Fig. 5B). This finding is consistent with results observed by CLSM. Interestingly, when the co-culture was plated on Todd Hewitt agar (THA) without antibiotics (total *S. mutans* count), the CFUs were equal to the mean of the mono-cultures. However, growth on the antibiotic selective media was more consistent with CLSM, exhibiting the doubling of total *S. mutans*, suggesting the use of antibiotic specific media is required to improve recovery and obtain accurate differential counts.

## DISCUSSION

Numerous association studies report that the presence of multiple *S. mutans* strains is associated with greater childhood caries (20–27), but studies exploring intraspecies interactions between *S. mutans* are limited. While interspecies studies are important to the study of dental caries, it is equally important to understand how intraspecies also contribute to caries. The oral cavity is a diverse ecological environment that is constantly under a variety of environmental stresses (38). Therefore, oral bacteria, especially *S. mutans*, are adept at adaptive horizontal gene transfer making for rich accessory genomes (18, 39–42). In this study, molecular biology approaches were applied to determine if the presence of multiple *S. mutans* strains are directly related to changes in measurable caries virulence traits.

In the initial population study for 10 children, it was notable that more of co-cultured biofilms for four-genotype children were significantly more acidic as compared to the children with two genotypes (Fig. 1A). This population-based initial approach indicates that the presence of more *S. mutans* can contribute to a significantly more acid environment enhancing cariogenicity. Furthermore, the use of clinical *S. mutans* with known prevalence data increases the clinical significance of this study (20).

Although G18 produces less biofilm biomass than the known cariogenic *S. mutans* UA159 strain, the biofilm analysis for Child 5 (C-232) suggests that G18 contributes most to the significantly acidic biofilm pH (Fig. 2C and D), while G09 significantly contributes to increased biofilm thickness (Fig. 3B). When co-cultured, these two strains have a mutually beneficial advantage in overall colonization *in vivo*, particularly for G09, leading to greater cariogenic potential over mono-cultures. Since both pHrodo and cascade blue are dextran-conjugated, and glucan was reportedly lower in G18 mono-culture, the actual increase in biofilm pH may be underestimated compared to G09 and mix (43). This finding is different than we previously reported for the representative library strain G18 (UAB-10), which produced significantly more glucan than UA159 (18).

The increase in cell density of *S. mutans* observed in the 2D biofilm imaging with Syto9 (Fig. 1B and 2C), together with the doubling effect in both CLSM analysis (Fig. 3) and *Drosophila* model (Fig. 5) for the co-cultures, supports that the presence of multiple *S. mutans* is mutually beneficial for the formation of biofilms. Although there was no significant difference in biofilm biomass (Fig. 2A), the increase in biofilm thickness and volume by CLSM (Fig. 3B and C) demonstrates the formation of more robust biofilms can improve colonization and persistence. It is possible that conditions that favor multiple *S. mutans* colonization may also favor *S. mutans* persistence, furthering caries development. Although, differential plate enumeration of CFUs per milliliter did not show a significant difference for post-biofilms analysis (Fig. S3), several factors may account for this difference, most notably the challenge of disrupting large biofilm aggregates efficiently.

Some studies have evaluated the spatial structure of interspecies oral biofilms using *in vitro* and *in vivo* models, demonstrating that oral biofilms form highly organized, spatially structured communities (44–48). The present study differs in its CLSM analysis of *S. mutans* intraspecies impact on spatial distribution and arrangement. Early colonization of *S. mutans* remains one of the best clinical indicators of ECC, especially in our study population (19). Given the high number of children within our study population with multiple *S. mutans* genotypes, it was worth investigating how *S. mutans* intraspecies interactions shape the early development of oral biofilm and the spatial landscape. The CLSM analysis supports that co-cultured *S. mutans* biofilms leads to increased biofilm thickness (Fig. 3B and D; Fig. S2; Movie S1). Furthermore, CLSM and time-lapsed imaging revealed that clinical *S. mutans* G09 and G18 each demonstrated unique phenotypes, occupied specific domains within the biofilm, and have no observable colocalization ( Fig. S4; Movie S2). The domed phenotype previously reported by Kim and Koo (45) was more similar to what was observed for the G09 phenotype in this study and differs significantly from the lawn phenotype observed for the G18 strain. Greater biofilm height from *S. mutans* G09 coupled with the fuller coverage of G18 contributes significantly to the surface area available for other organisms to attach, increasing overall cariogenic potential of co-cultured *S. mutans* biofilms.

Although *S. mutans* UA159 has been widely studied and is known to be cariogenic in a rat model, it is crucial to understand that other clinical *S. mutans* can contribute to caries in a very different manner than UA159. It has been well documented that clinical *S. mutans* can differ considerably in their cariogenic phenotypes (11, 39, 49). It is important to note that biofilm experiments should be tested with other clinical strains, including those showing inhibition against other *S. mutans,* to provide a more comprehensive understanding of intraspecies interactions among clinical isolates. Future studies are planned to evaluate how intraspecies *S. mutans* interactions impact the spatial landscape in polymicrobial models.

The time-lapsed data included in this study provide valuable new information on how acid is formed (Fig. 5). The observed bi-phasic effect in the pHrodo suggests *S. mutans* may experience a period of equilibration in response to the initial acid production, possibly through the activation of genes related acid tolerance response due to acid stress (11, 50, 51). Further study is needed to elucidate this phenomenon.

The finding that the presence of multiple *S. mutans* within the first year of detection is significantly associated with having caries is informative, indicating the presence of multiple *S. mutans* is linked and likely contributes to early onset of ECC within the first year of colonization. Among caries-free children, the majority (86%) did not show persistent colonization of *S. mutans*, that is, *S. mutans* strains were acquired and lost, or colonized in subsequent periods with a different strain, which were later lost. These children would make excellent subjects for subsequent host immunology and microbiome studies to determine why *S. mutans* was unsuccessful in colonizing the oral cavity of these children.

In summary, when multiple *S. mutans* strains G09 and G18 are present, there are significant increases in cariogenic properties including colonization, surface area coverage, cell density, and acid production. The high prevalence of G09 and G18 *S. mutans* genotypes in our high caries risk population (20) and the large percentage of children with multiple strain types within the first year of detection of *S. mutans* suggest that having multiple genotypes, particularly *S. mutans* G09 and G18, is a strong risk factor for early childhood caries.

## MATERIALS AND METHODS

### Bacterial strains

*S. mutans* isolates from 78 African-American children (approximately 1 year of age at baseline and followed until 6 years) from a previous 8-year longitudinal study in a high-risk caries population were used. Isolates were obtained from either plaque or saliva as previously described (20). This population was selected due to lack of water fluoridation. Furthermore, we reported 65.8% of children in this population had ECC by age 4 (52). From over 14,000 bacterial isolates initially evaluated, a total of 34 representative genotypes were determined by rep-PCR and confirmed by whole genome sequencing as genetically distinct in this population (18, 20). Since longitudinal data of acquisition of *S. mutans* genotypes were available, this provides a key advantage in the present study since a patient-based model was used (i.e., actual strains from specific caries-active children were used).

Previously, we reported an overview analysis of single vs multiple genotypes within this population with details on collection methods and rep-PCR analysis (20, 53). For the present study, we provide additional analysis on the relationship of having multiple *S. mutans* at initial detection of *S. mutans* or within the first 6-month visit after initial detection with caries (yes/no) for cohort 2 (approximate age 1 year at baseline) who had at least seven isolates (*n* = 78) analyzed by rep-PCR. Caries was defined for a child with a decayed, missing, or filled surfaces (DMFS) > 0 at any time during the previous study (6 years) (54). We selected this time point for caries analysis since the time to visual demineralization can take 1–2 years following bacterial colonization.

Preliminary analysis of biofilms was performed with *S. mutans* isolates from 10 caries-active children (five children presenting two genotypes, five children presenting four genotypes during the previous study) (Table 2). One child (C-232, Child 5) with two genotypes was selected for further study. This child presented with *S. mutans* genotypes G18 and G09, the two most prevalent genotypes observed in the larger epidemiological study of this high-caries risk population (20). *S. mutans* UA159 was used as a positive control on all experiments.

**TABLE 2:** List of bacterial strains, plasmids, and primers used in this study

Strain, plasmid, or primer	Description	Sequence	Source
Strains			
	*Streptococcus mutans* clinical isolates from Uniontown epidemiological study		(20)
Sm232-G09	G09 *S. mutans* clinical isolate parent strain		This study
Sm232-G18	G18 *S. mutans* clinical isolate parent strain		This study
Sm232-G09 mcherry	G09 *S. mutans* clinical isolate modified with mCherry-Ery resistant plasmid		This study
Sm232-G09 GFP	G09 *S. mutans* clinical isolate modified with GFP-Kan resistant plasmid		This study
Sm232-G18 GFP	G18 *S. mutans* clinical isolate modified with GFP-Kan resistant plasmid		This study
Sm232-G18 mcherry	G18 *S. mutans* clinical isolate modified with mCherry-Ery resistant plasmid		This study
UA159	*S. mutans* control reference strain		(55)
UA159 GFP	*S. mutans* UA159 with GFP-Kan resistant plasmid		(56, 57)
Plasmids			
pALH134	Promoterless *aphA3* Kan resistant cassette		L. Honeyman
pVPT-GFP	*Escherichia coli*-*Streptococcus mutans* shuttle vector and expression plasmid Ery resistant		(58)
p8912Cherry	mCherry fluorescent plasmid		(59)
Primers			
pVPT SacI F	Inverse PCR primer for pVPT plasmid forward	GATCATGAGCTCTTCTATGAGTCGCTTTTGTAA	This study
pVPT SacI R	Inverse PCR primer for pVPT plasmid reverse	GATCATGAGCTCGTAATCACTCCTTCTTAATTACAAAT	This study

### Mutant constructions

To determine strain prevalence within the mixed biofilm matrix and biofilm spatial arrangement by confocal microscopy, the *S. mutans* G18 and G09 strains from Child 5 (C-232) were genetically modified with plasmids carrying different fluorescent proteins and antibiotic resistance genes (green fluorescent protein pVPT + Kan^R^ and red fluorescent protein mCherry + Ery^R^, respectively) (Table 2) (31, 59, 60). To make the pVPT-GFP + Kan^R^ plasmid, the erythromycin gene of the pVPT + GFP plasmid was removed using reverse PCR with the pVPT SacI forward and reverse primers (Table 2). Next, PCR ligation mutagenesis was used to insert a nonpolar kanamycin resistance cassette obtained by enzyme digestion from pALH124 using SacI restriction enzyme. Ligation product was transformed into *Escherichia coli* cells and kanamycin-resistant transformants were PCR screened to confirm mutation. Plasmids were confirmed using sequencing prior to transformation into *S. mutans* strains. Antibiotics were used in the following concentrations: 1 mg/mL kanamycin and 10 µg/mL erythromycin for *S. mutans* and 50 µg/mL kanamycin for *E. coli*.

### Biofilm analysis

*In vitro* static biofilm analysis was performed as previously described (18). Briefly, *S. mutans* isolates were plated on THA from frozen stocks for secondary pure culure and grown anaerobically (10% hydrogen, 10% carbon dioxide, 80% nitrogen) for 48 hours at 37°C then sub-cultured in Todd Hewitt broth (THB) to optical density OD_600_≈ 0.5 before diluting 1:1,000 in THB + 1% sucrose. Samples were prepared for individual mono-cultures and co-culture (mix of two or four *S*. *mutans* together). For single cultures, 5 µL of sub-culture was added to 5 mL THB + 1% sucrose for biofilm set-up. For co-cultured mixed samples, 10 µL of each sub-cultured strain was mixed, then 5 µL of this mixture was used for biofilm set-up, such that all biofilms were inoculated with comparable volumes of *S. mutans*. For the pH assay, pHrodo red or cascade blue dextran-conjugated probes (Molecular Probes, Invitrogen, Carlsbad, CA) were added 1:1,000 prior to biofilm set-up to monitor pH and glucan production, respectively. A 96-well flat-bottom microplate was used with 200 µL per well. All biofilm plates were incubated for 16 hours in 5% CO_2_ incubator at 37°C under static conditions. Only one biological replicate was performed in the initial analysis for the 10 children (triple technical replicates), as the purpose was to demonstrate a trend within the overall population. For all subsequent analysis, C-232 (Child 5) was used with a minimum of three biological replicates and three technical replicates each were performed.

Biofilms analysis was performed initially to assess biomass (crystal violet assay), intracellular IPS (to assess glycogen by iodine assay), and pH (pHrodo/cascade blue/Syto9) for the 10 children as described (13, 18, 32, 61–63). OD_562_ and OD_520_ were measured for biomass and IPS, respectively. To determine biofilm pH, wells were manually surveyed and 2D representative images collected at 10× using a fluorescent microscope (Nikon TE2000-E inverted scope). ImageJ was used to calculate mean values of the fluorescence to determine pH (pHrodo), glucan (cascade blue), and cell density (Syto9).

### CLSM analysis

Biofilms were prepared as described above, except in eight-well ibidi-treated slides (ibidi, Fitchburg, WI) with 300 µL per well in duplicate wells. Overnight biofilms were washed three times with 1× phosphate buffered saline (PBS). Confocal microscopy was performed with three biological replicates, with a minimum of five image sites collected per sample.

Biofilms were imaged on a Zeiss LSM 880 or LSM 980 laser scanning confocal through a 40 × 1.2 LD LCI Plan-Apochromat water immersion lens and excitation and emission parameters suggested by the manufacturer for mCherry, GFP, and cascade blue. Image analysis of average intensity for all three channels in volume segmentations was performed in Bitplane Imaris v.10.0.1. The average height of the biofilms was assessed from orthogonal projections spanning the full depth of the field of view.

### Time-lapsed biofilms analysis

To examine the biofilm architecture formation over time, biofilms were prepared as described above for CLSM and imaged every hour for 24 hours using a Zeiss Cell Discoverer7 (CD7) instrument using a 50× PlanApp 1.2NA Water immersion lens in combination with a 0.5 tube lens and detection on a Zeiss Axiocam 506 m camera at a pixel resolution of 0.181 µm per pixel and a field-of-view of 320 µm by 285 µm. Imaging environment was kept at a constant of 37°C and 5% CO_2_. A 40 µm z-stack at 1 µm interval was acquired every hour for 24 hours with two fluorescence channels (mCherry and GFP) as needed, as well as a brightfield image contrasted through oblique illumination. The fluorescence and brightfield channels were processed separately for each position over time. Background in fluorescence channels was equalized and subtracted using a rolling ball algorithm, and channel crosstalk was reduced using linear unmixing. The bottommost six slices of the fluorescence channels were orthogonally projected for maximum intensity in each pixel and overlaid with the most contrasted transmitted image from the z-stack prior to export into a movie file.

To investigate the formation of biofilm pH over time, *S. mutans* G09 GFP and G18 GFP were used with pHrodo red dextran-conjugated probe to determine pH with the methods described above with imaging performed every hour for 30 hours. All 24- to 30-hour experiments were performed in duplicate on two independent experiments with three to four sites per well, and cascade blue was not used in these experiments.

### Differential plating

To determine the distribution of G18 and G09 within the single and mixed biofilms, differential plating on specific antibiotic agars was used. “Pre-plate” was from the original culture used to inoculate the biofilms to determine if starting amounts of *S. mutans* were accurate and comparable between single and mixed cultures. “Post-plate” was from 16-hour biofilm, washed three times with sterile 1× PBS, manually dislodged, and briefly sonicated. All samples were serially diluted, then 100 µL plated in duplicate on THA with respective antibiotics. CFUs (CFUs per milliliter) were manually enumerated.

### *Drosophila* colonization model

A *Drosophila melanogaster* model was used to determine colonization *in vivo* for the single and co-cultured *S. mutans*. This model has been commonly used to determine colonization of *S. mutans* and other bacteria (31–37). Flies were orally infected by modified capillary feeding system (Fig. S6) (64). Seven male Canton-S flies (1–3 days old) were treated with antibiotics (Jazz Mix Drosophila food with erythromycin, vancomycin, and ampicillin 50 µg/mL) for 2 days. Fresh overnight (first feeding) and mid-log sub-cultured *S. mutans* (OD_600_ ≈ 0.5, second feeding, 8 hours after first) were used. This twice-daily feeding appeared to minimize biofilm formation within the capillaries and allowed continued access to the bacterial/sucrose food. Respective antibiotics were used to maintain plasmids in all cultures. Aliquots of 1 mL for each mono-culture and 500 µL for of each culture for mixed sample (total volume 1 mL) were centrifuged at 10,000 rpm for 3 min, and bacterial pellets were washed two times with 1× sterile PBS to remove residual media and antibiotics (to improve consistency of CFU counts). Harvested cells were resuspended in 100 µL 5% sucrose and 0.5 µL of suspensions were added to calibrated micro-capillary tubes (Miles Scientific, Newark, DE) inserted via pipette tips through cotton vial caps. Flies were incubated at room temperature and protected from direct light. Flies were fed by capillary feeding method for 3 days. Flies were then rendered unconcious in a −20°C freezer for 1 hour. Next, five flies were briefly sterilized with 70% ethanol, washed three times with sterile PBS, and pulverized in 200 µL sterile PBS. Serial dilutions of homogenate were spread on respective THA with antibiotics. Plates were incubated 48 hours at 37°C in 5% CO_2_ and CFUs were enumerated.

### Statistical analysis

Fisher’s exact test was used for association analysis of multiple *S. mutans* with caries. Statistical analysis was performed using either the Student’s *t*-test or one way analysis of variance with Tukey *ad hoc* with statistical significance set to (*P* < 0.05).

## SUPPLEMENTAL MATERIAL

Fig. S1 msphere.00778-23-s0001.pdf Biomass and IPS for population.

Fig. S2 msphere.00778-23-s0002.pdf Representative orthogonal views in CLSM.

Fig. S3 msphere.00778-23-s0003.pdf Differential plate counts for Pre and Post biofilms.

Fig. S4. msphere.00778-23-s0004.pdf Representative 5-h time-lapse comparison of early biofilm formations.

Fig. S5 msphere.00778-23-s0005.pdf Representative biofilms using oblique or fluorescent imaging for 12-h time lapse.

Fig. S6 msphere.00778-23-s0006.pdf Modified capillary feeding method for *Drosophila*.

Movie S1 msphere.00778-23-s0007.mp4 CSLM mix biofilm architecture. Red, G09; Green, 18; blue, glucan.

Movie S2 msphere.00778-23-s0008.mp4 Oblique and fluorescent 12-h time-lapse imaging.

ASM does not own the copyrights to Supplemental Material that may be linked to, or accessed through, an article. The authors have granted ASM a non-exclusive, world-wide license to publish the Supplemental Material files. Please contact the corresponding author directly for reuse.
