# Supplementary material for: Intraspecies interactions of Streptococcus mutans impact biofilm architecture and virulence determinants in childhood dental caries
Source: mSphere. 2024 Jul 11;9(7):e00778-23. doi: 10.1128/msphere.00778-23 (PMC11288028; doi:10.1128/msphere.00778-23)
Supplement: Fig. S1 — Biomass and IPS for population. [file msphere.00778-23-s0001.pdf]

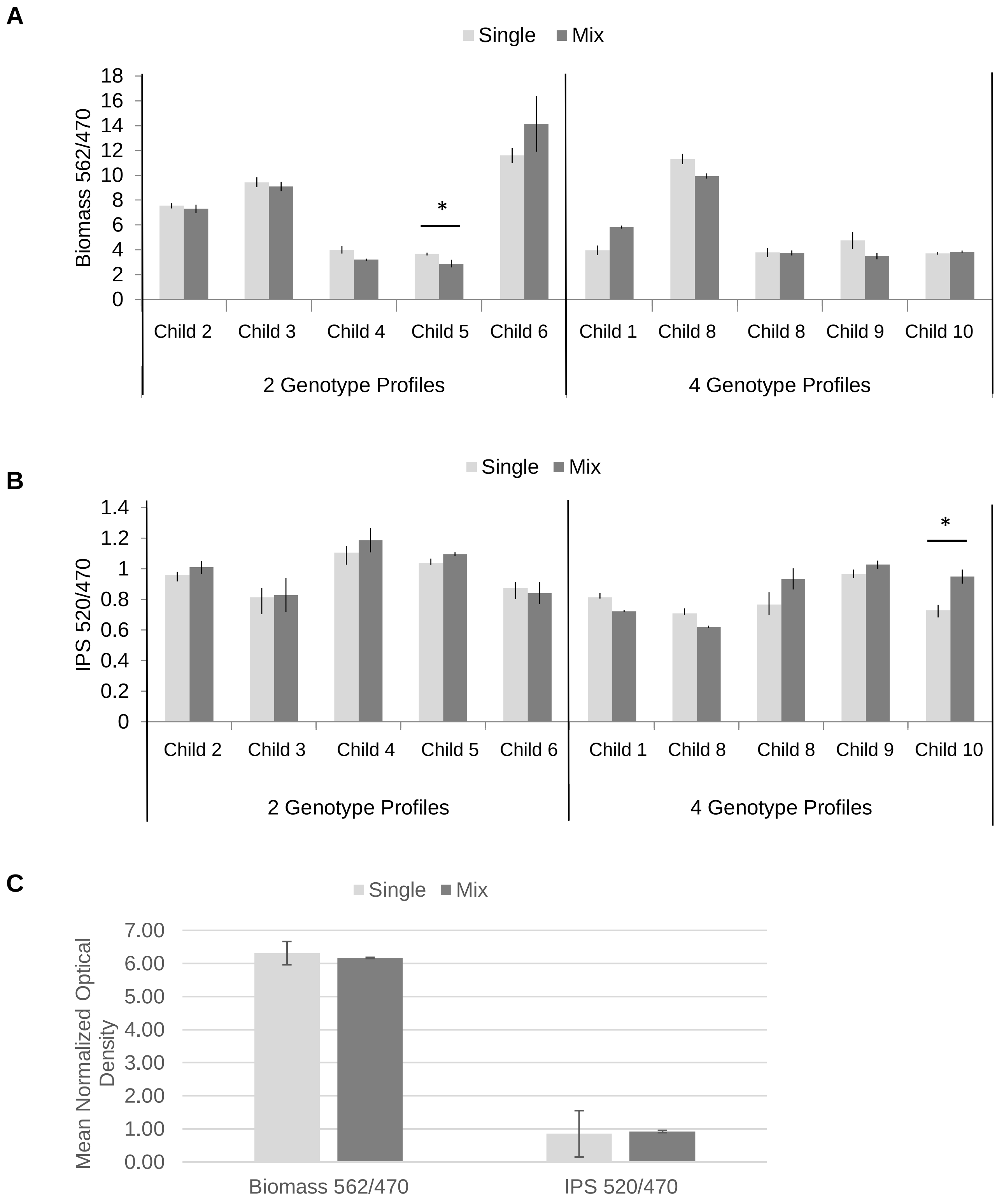
A
Single
Mix
Biomass 562/470
18
16
14
12
10
8
6
4
2
0
*
Child 2
Child 3
Child 4
Child 5
Child 6
Child 1
Child 8
Child 8
Child 9
Child 10
2 Genotype Profiles
4 Genotype Profiles
B
Single
Mix
IPS 520/470
1.4
1.2
1
0.8
0.6
0.4
0.2
0
*
Child 2
Child 3
Child 4
Child 5
Child 6
Child 1
Child 8
Child 8
Child 9
Child 10
2 Genotype Profiles
4 Genotype Profiles
C
Single
Mix
Mean Normalized Optical Density
7.00
6.00
5.00
4.00
3.00
2.00
1.00
0.00
Biomass 562/470
IPS 520/470

FIG S1 Biofilm biomass and intracellular polysaccharide (IPS) for mono- and co-cultured *S. mutans* for 10 individual children with either two or four *S. mutans* genotypes shows no statistical difference on average for the population. (A) Mean normalized *S. mutans* biofilm biomass assessed by crystal violet assay. (B) Mean normalized *S. mutans* biofilm IPS assessed by iodine assay show no significant difference on average for the population although 60% trended higher in the co-cultured biofilms. For simplicity, the mean of single cultures are shown**.** (C) Mean normalized *S. mutans* biomass and IPS for all 10 children combined support no statistical difference. N=1 with 3 technical replicates to observe the trend in the population. Standard error bars shown. * $P<0.05$, ** $P<0.01$, ***$P<0.001$
