# Supplementary material for: Intraspecies interactions of Streptococcus mutans impact biofilm architecture and virulence determinants in childhood dental caries
Source: mSphere. 2024 Jul 11;9(7):e00778-23. doi: 10.1128/msphere.00778-23 (PMC11288028; doi:10.1128/msphere.00778-23)
Supplement: Fig. S2 — Representative orthogonal views in CLSM. [file msphere.00778-23-s0002.pdf]

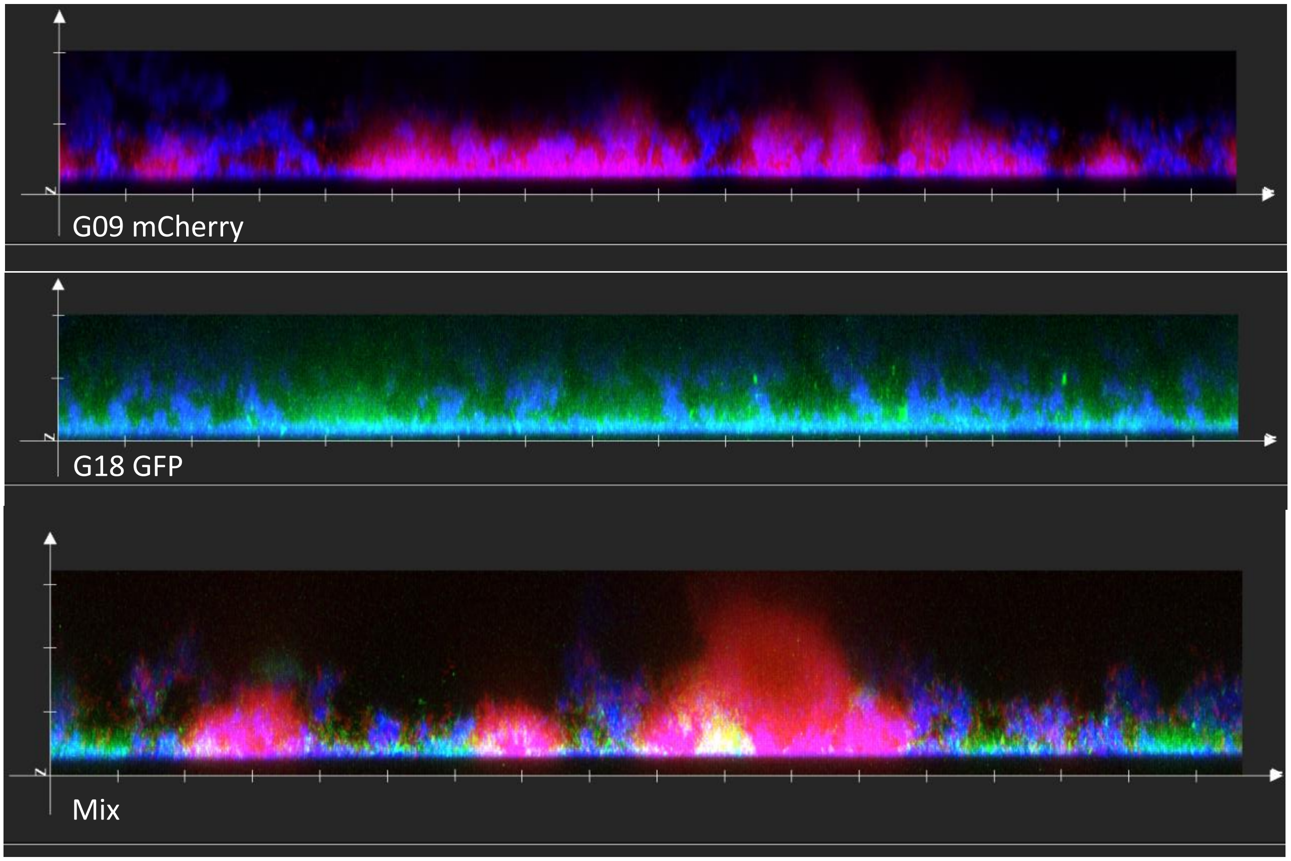


**FIG S2** Representative CLSM Orthogonal views for mono- and co-cultured biofilms illustrates the increased height in G09 biofilm aggregates towers (shown in red) contributing to increase biofilm thickness in co-culture (Mix) versus single culture biofilms. Each tick on y-axis is 5 μm. *S. mutans* G09 (red) dwarfs G18 (green) and glucan (cascade blue) in the co-cultured biofilms.
