# Supplementary material for: Intraspecies interactions of Streptococcus mutans impact biofilm architecture and virulence determinants in childhood dental caries
Source: mSphere. 2024 Jul 11;9(7):e00778-23. doi: 10.1128/msphere.00778-23 (PMC11288028; doi:10.1128/msphere.00778-23)
Supplement: Fig. S3 — Differential plate counts for Pre and Post biofilms. [file msphere.00778-23-s0003.pdf]

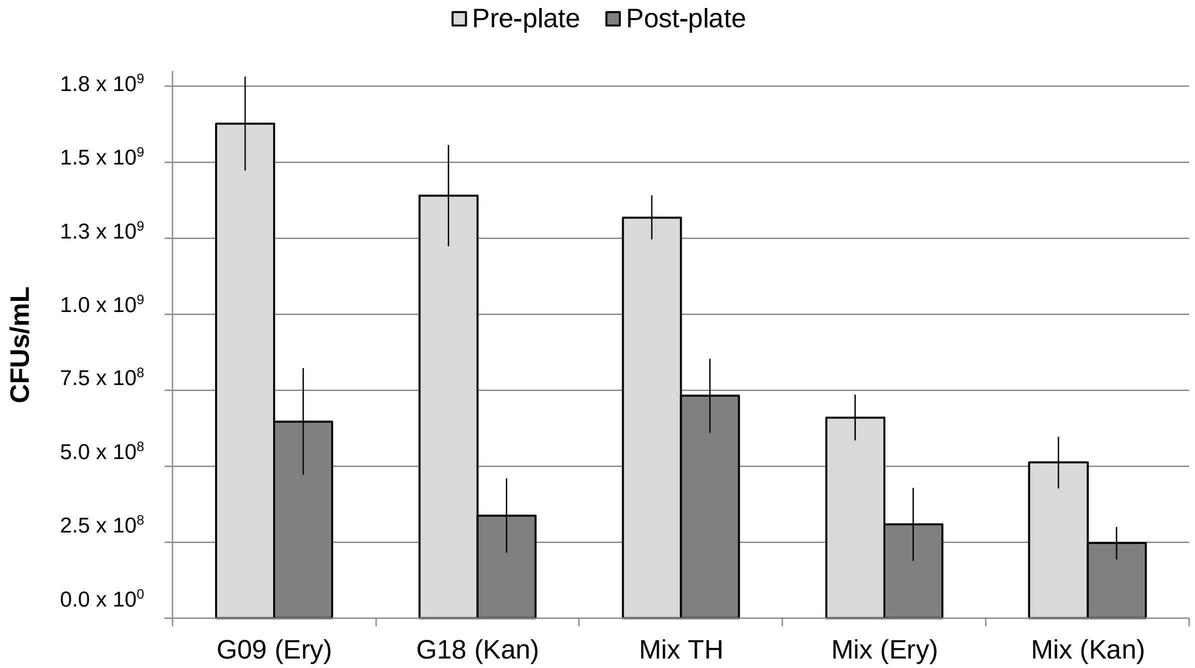

FIG S3 Differential plating of *S. mutans* for Pre- and Post- biofilms. Data supports that both mono- and co-cultured biofilms were inoculated with comparable amounts of *S. mutans*. The Mix (co-culture) was comprised of equal volumes of G09 and G18 from Child 5 (C-232). Mix was plated on Todd Hewitt Agar (THA) for total *S. mutans* count and on THA with antibiotics to quantify each strain within the Mix. Erythromycin (Ery) and kanamycin (Kan) were used for G09 and 18, respectively. N=4 with 2 technical replicates each.
