# Supplementary material for: Intraspecies interactions of Streptococcus mutans impact biofilm architecture and virulence determinants in childhood dental caries
Source: mSphere. 2024 Jul 11;9(7):e00778-23. doi: 10.1128/msphere.00778-23 (PMC11288028; doi:10.1128/msphere.00778-23)
Supplement: Fig. S4. — Representative 5-h time-lapse comparison of early biofilm formations. [file msphere.00778-23-s0004.pdf]

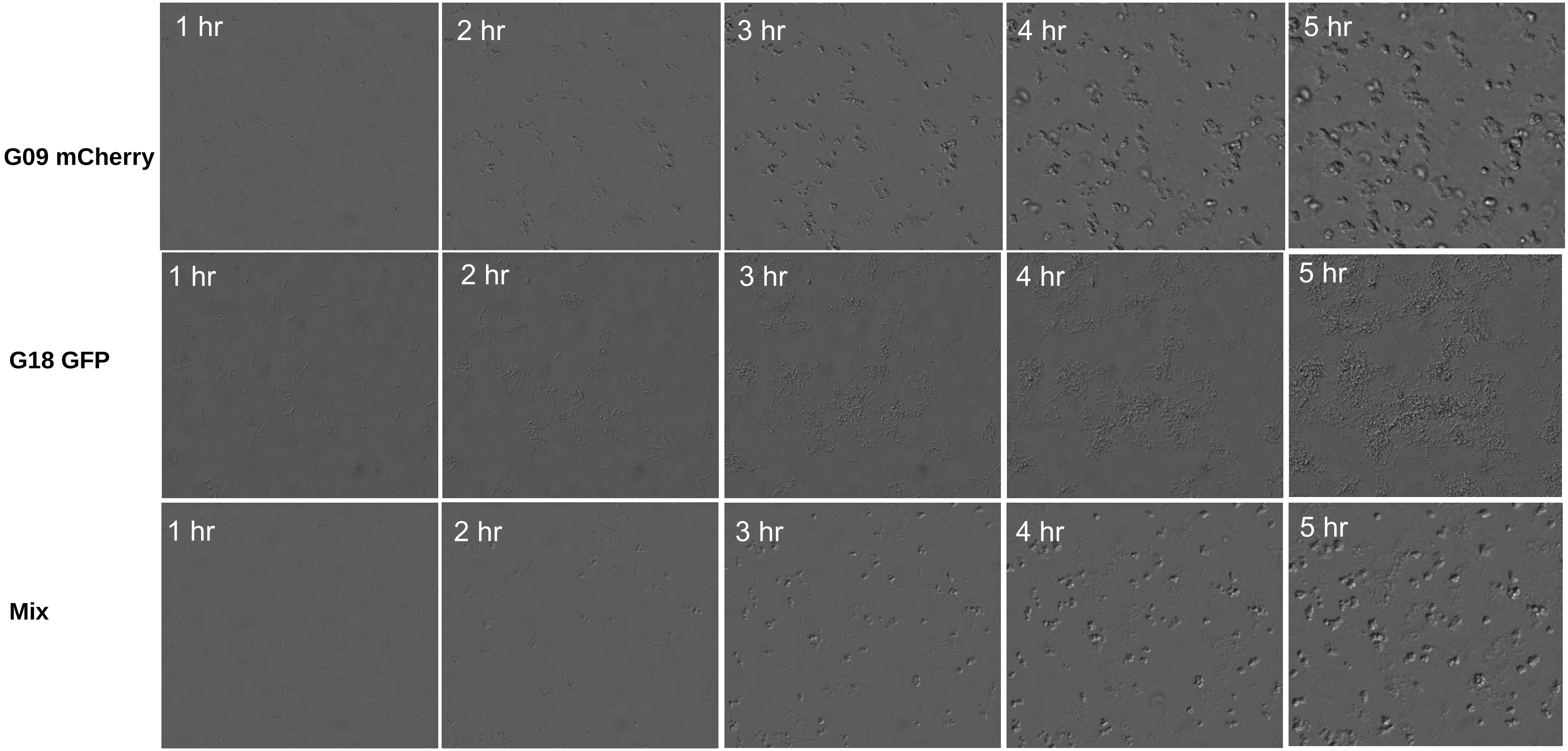

FIG S4 Representative contrasted oblique illumination comparison of biofilm formation of single culture versus co-culture (Mix) *S. mutans* G09 and G18 from Child 5 (C-232). *S. mutans* biofilms shows distinct biofilm phenotypes as early as the first 5 hours of growth. *S. mutans* G09 (denser aggregates) and G18 (thinner “fish-nets”) are clearly distinguishable in the co-culture over time. Biofilms were grown in 5% carbon dioxide at 37°C using Zeiss CD7 Cell Discoverer microscope with 50X PlanApp Water immersion lens. N=3 independent experiments performed in duplicate with 3-4 image sites per well.
