# Supplementary material for: Intraspecies interactions of Streptococcus mutans impact biofilm architecture and virulence determinants in childhood dental caries
Source: mSphere. 2024 Jul 11;9(7):e00778-23. doi: 10.1128/msphere.00778-23 (PMC11288028; doi:10.1128/msphere.00778-23)
Supplement: Fig. S5 — Representative biofilms using oblique or fluorescent imaging for 12-h time lapse. [file msphere.00778-23-s0005.pdf]

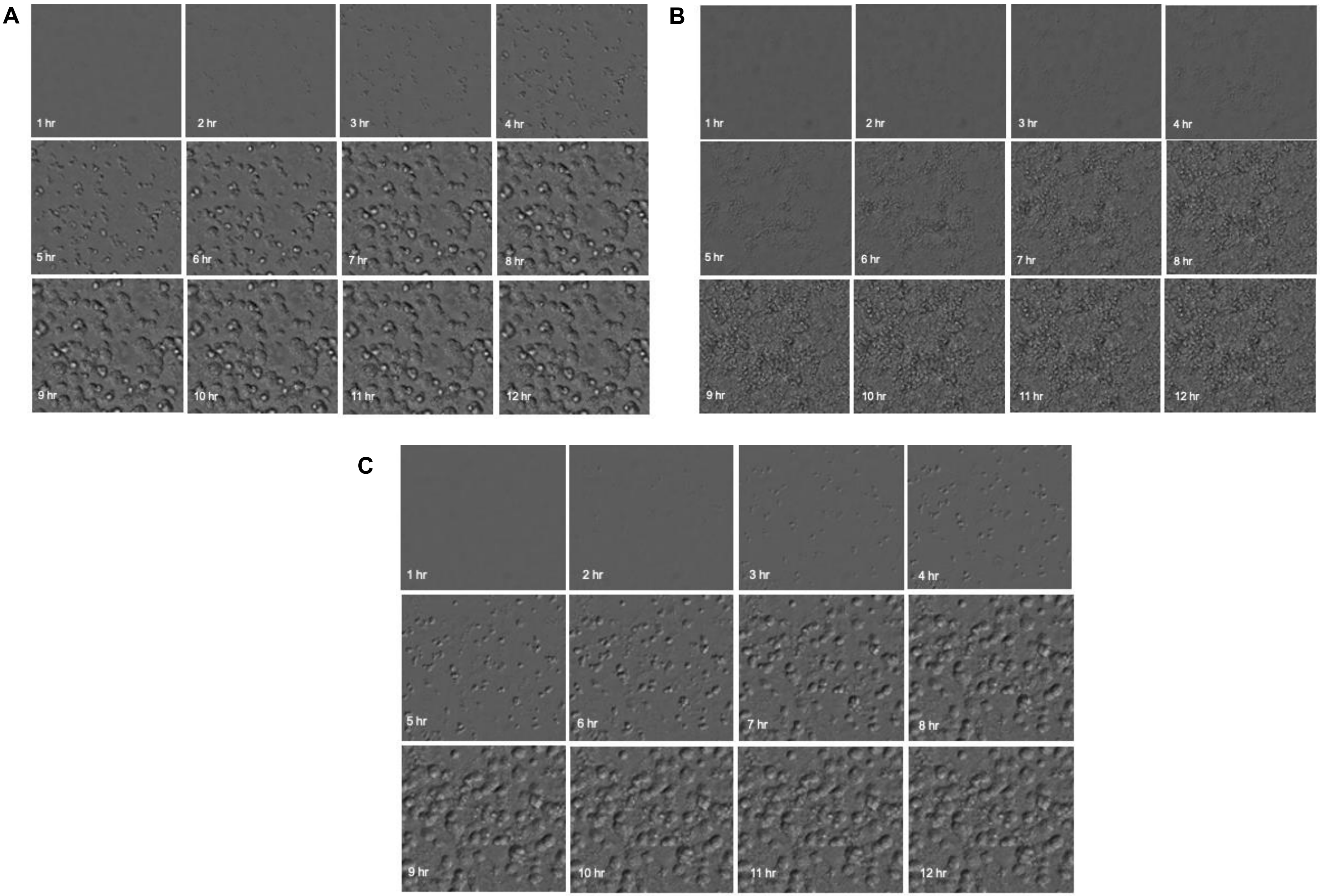
A
1 hr
2 hr
3 hr
4 hr
5 hr
6 hr
7 hr
8 hr
9 hr
10 hr
11 hr
12 hr
B
1 hr
2 hr
3 hr
4 hr
5 hr
6 hr
7 hr
8 hr
9 hr
10 hr
11 hr
12 hr
C
1 hr
2 hr
3 hr
4 hr
5 hr
6 hr
7 hr
8 hr
9 hr
10 hr
11 hr
12 hr

D

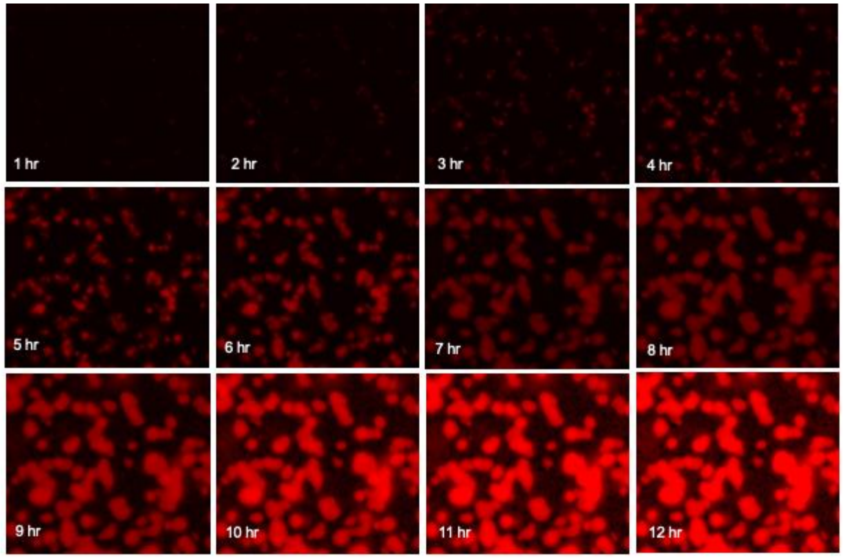
1 hr
2 hr
3 hr
4 hr
5 hr
6 hr
7 hr
8 hr
9 hr
10 hr
11 hr
12 hr

E

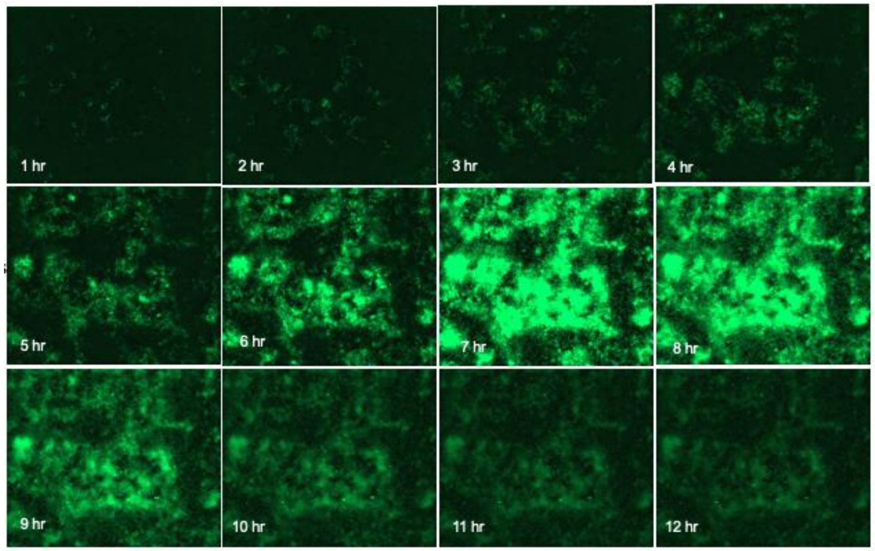
1 hr
2 hr
3 hr
4 hr
5 hr
6 hr
7 hr
8 hr
9 hr
10 hr
11 hr
12 hr

F

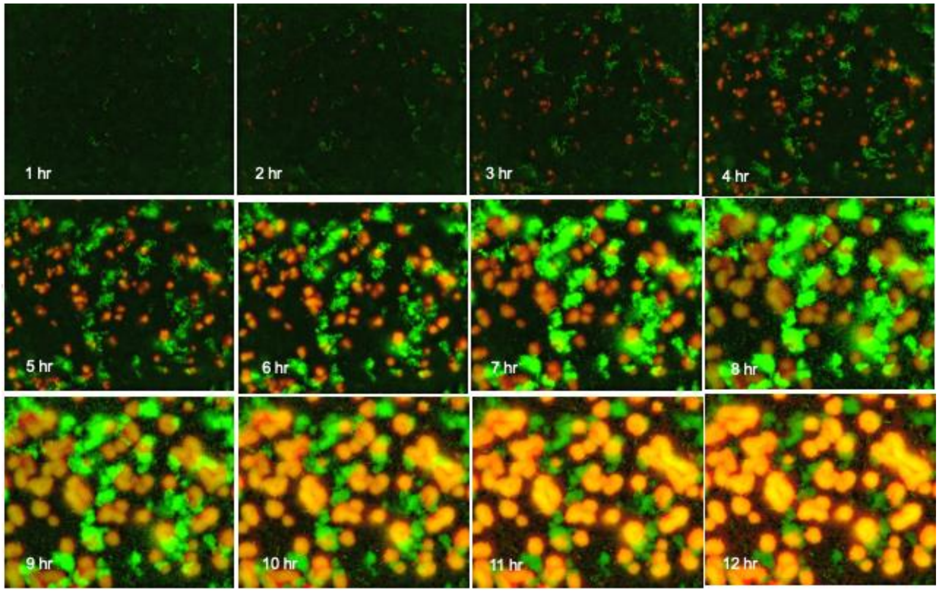
1 hr
2 hr
3 hr
4 hr
5 hr
6 hr
7 hr
8 hr
9 hr
10 hr
11 hr
12 hr

FIG S5 Representative contrast oblique illumination and fluorescence imaging for 12-hour time-lapse for *S. mutans* biofilm formation for mono-culture and co-cultures. First 12 hours contrast oblique illumination for (A) G09 mono-culture, (B) G18 mono-culture and (C) co-culture G09 and G18 mix show distinguishable biofilm architecture over time. First 12 hours fluorescence imaging for (D) G09 mono-culture with mCherry red, (E) G18 mono-culture with green fluorescent protein, and (F) co-culture G09 and G18 mix demonstrates mCherry increased intensity and GFP decreased intensity. In mono and co-cultured biofilm G18 has a peak intensity around 7-8 hours while G09 predominates later. Fluorescent imaging brightness increased 20% for all images. *S. mutans* G09 and G18 from Child 5 (C-232).
