# Supplementary material for: Intraspecies interactions of Streptococcus mutans impact biofilm architecture and virulence determinants in childhood dental caries
Source: mSphere. 2024 Jul 11;9(7):e00778-23. doi: 10.1128/msphere.00778-23 (PMC11288028; doi:10.1128/msphere.00778-23)
Supplement: Fig. S6 — Modified capillary feeding method for Drosophila. [file msphere.00778-23-s0006.pdf]

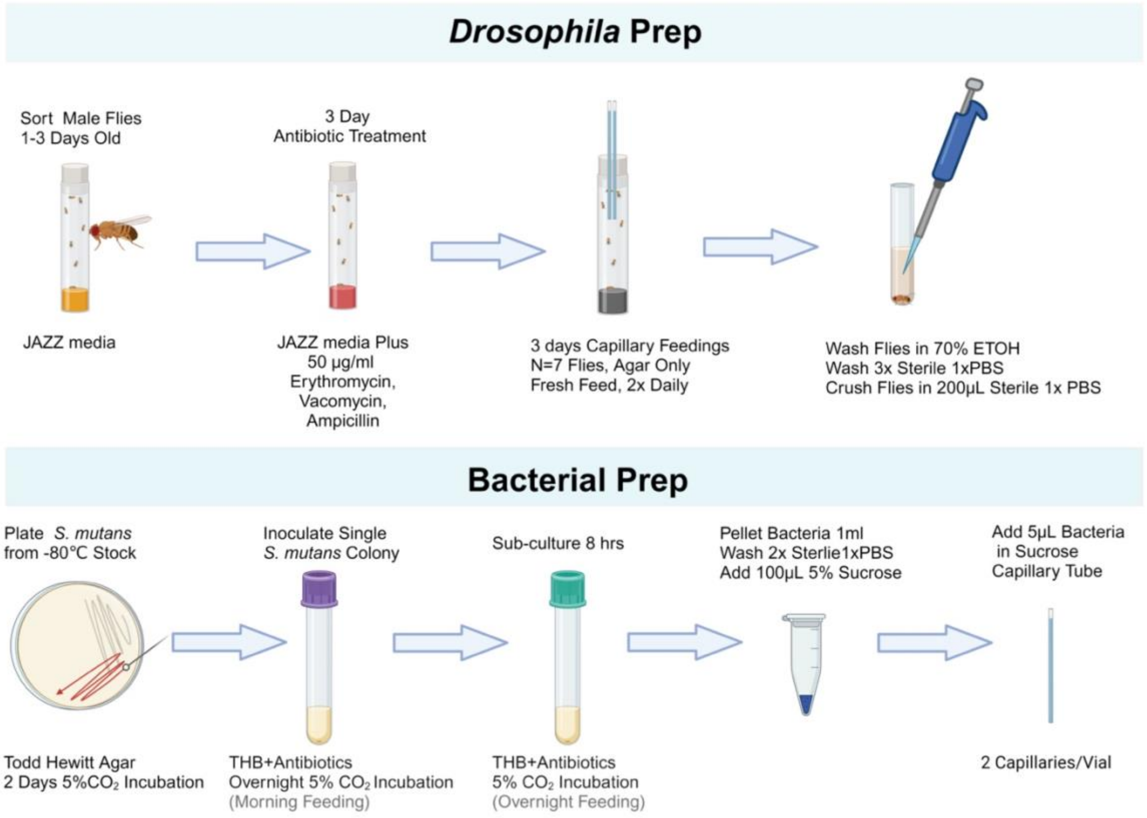

FIG S6 Modified Capillary Feeding method for *Drosophila*. Modified based on Diegelman et al. 2017 *J Vis Exp*. This method uses commercially available non-toxic cellulose acetate vial closures with pipette tips inserted for easy insertion and removal of feeding tubes. Pipette tips and feeding tubes were inserted to just below the bottom surface of the cellulose acetate plugs to improve access of flies to feeding tubes. Modified processing of bacterial cells including washing bacterial cell pellets to remove residual antibiotics and twice-daily feeding with fresh cultures to improve colonization of *S. mutans*. In our model, 7 flies were used per vial (this allowed 2 extra flies in case of death) and 5 flies were used to homogenize and plate for CFU recovery on selective media. Image generate with BioRender.
